# German Million Children Cohort: a historical birth cohort based on claims data to investigate the impact of immunisation and other early life factors on the risk of cancer and other diseases in childhood – cohort profile

**DOI:** 10.1136/bmjopen-2025-113411

**Published:** 2026-03-26

**Authors:** Lara Kim Brackmann, Loviisa Mulanje, Sophie Langbein, Bianca Kollhorst, Ulrike Haug, Wolfgang Ahrens, Rajini Nagrani, Manuela Marron

**Affiliations:** 1 Epidemiological Methods and Etiological Research, Leibniz Institute for Prevention Research and Epidemiology - BIPS, Bremen, Germany; 2 Faculty of Human and Health Sciences, University of Bremen, Bremen, Germany; 3 Statistical Methods in Epidemiology, Leibniz Institute for Prevention Research and Epidemiology - BIPS, Bremen, Germany; 4 Faculty of Mathematics and Computer Science, University of Bremen, Bremen, Germany; 5 Clinical Epidemiology, Leibniz Institute for Prevention Research and Epidemiology - BIPS, Bremen, Germany

**Keywords:** Vaccination, Child, Paediatric infectious disease & immunisation, Cancer, INFECTIOUS DISEASES

## Abstract

**Abstract:**

**Purpose:**

As a historical register-based birth cohort, the *German Million Children Cohort* was set up using data from the *German Pharmacoepidemiological Research Database (GePaRD*), a health insurance database covering ~20% of the German population. The first cohort project aims to investigate the impact of *Prenatal and Childhood Immunization and the Risk of Childhood Cancer* project.

**Participants:**

The cohort includes all newborns in *GePaRD* from 2004 to 2018 and is followed until occurrence of childhood cancer, death, the end of continuous insurance or currently available data years (31 December 2022). Information on vaccinations and confounders is collected from the inpatient and outpatient settings. For most children, information on vaccination exposure during pregnancy can be obtained through mother-child linkage.

**Findings to date:**

The cohort includes 2 023 613 children with a maximum follow-up of 18 years. This results in 17 752 995 person-years at risk and 3410 cases of childhood cancer. Maternal linkage was possible for 78.3% of children. The vaccination coverage for both nationally recommended and other vaccines was assessed in the cohort. By age 30 months, 38.9% of children received all recommended vaccines, 52.4% received incomplete vaccinations and 8.8% remained unvaccinated. Regarding further characteristics, 57.6% of children were from families with higher education, 20.3% were hospitalised due to a severe infection during follow-up and 74.5% received at least one antibiotic prescription.

**Future plans:**

While our first study with this historical cohort is dedicated to the question of whether immunity to childhood infections that are covered by the recommended baseline vaccination scheme reduces the risk of childhood cancer, the cohort will offer further opportunities. This includes the evaluation of additional factors potentially influencing the risk of cancer and other diseases in childhood, as well as an extension of the inclusion period beyond 2018 to also include children born during the COVID-19 pandemic.

**STRENGTHS AND LIMITATIONS OF THIS STUDY**

- The large size of this historical birth cohort enables precise effect estimates, making it a promising approach to investigate the impact of immunisation on childhood cancer. With insured individuals from all social groups and all geographical regions of Germany, the cohort represents the general population quite well, ensuring the generalisability of the results.
- The extensive data coverage of the *German Million Children Cohort*, with up to 18 years of follow-up from 2004 to 2022, enables long-term follow-up of the study population and the ascertainment of incident cancers during the early life period, based on clinical data.
- By linking lifetime information of children and their mothers, the study can additionally explore the impact of prenatal exposures on the risk of childhood cancer.
- Exposure to potential medical risk factors is identified from routinely collected claims data directly reflecting clinical records. Using this database prevents non-response and recall bias. Additionally, confounding factors, such as specific comorbidities or medications as well as socioeconomic status, are considered.
- A limitation of the *German Million Children Cohort* is that the used healthcare database only provides limited information on environmental exposures and behaviour.

## Introduction

 Every year in Germany, around 2200 children and adolescents aged 0–18 years are diagnosed with cancer,^1^ hereafter referred to as childhood cancers. This incidence corresponds to an age-standardised rate of 17.0 cases per 100 000 children. Between 1980 and 2018, the *German Childhood Cancer Registry* documented 66 859 new cases.^1^ The most common diagnoses were leukaemia (29.7%), central nervous system (CNS) tumours (including brain tumours; 23.6%) and lymphomas (15.3%), with median ages at diagnosis being 5 years for leukaemia, 7 years for CNS tumours and 13 years for lymphomas.^1^ Malignant brain tumours and leukaemia are among the leading causes of death in children under 15 years in Germany.^2^ Currently, about 82% of affected children in Germany survive for at least 15 years post diagnosis.^1^ Childhood cancers often lead to reduced quality of life^3 4^ and long-term sequelae such as second primary neoplasms,^5^ infertility,^6^ neurocognitive impairment^7^ and chronic cardiovascular or pulmonary diseases.^8^,^10^

The risk factors for childhood cancer remain largely unclear and vary by tumour types due to distinct histological characteristics and pathogenetic mechanisms.^11 12^ About 5%–10% of common childhood cancers are attributed to rare genetic syndromes with a high risk of cancer,^13^,^15^ while commonly occurring polymorphisms are associated with only a small increase in childhood cancer risk.^16^,^20^ Established environmental risk factors are ionising radiation for leukaemia^21^,^27^ and certain chemical exposures, such as benzene and cytostatic drugs for acute myeloid leukaemia in particular.^14 15^ Again, these single environmental exposures account for only a small fraction of new childhood cancer cases, and most affected children have not been exposed. Consequently, it is hypothesised that childhood cancer may result from the interplay of multiple prenatal and postnatal factors.^14 28^

Research increasingly highlights the immune system as a critical factor in cancer development.^29^ Greaves’ immune theory^30^ proposes that insufficient immune stimulation in the first year of life may increase the risk of acute lymphoblastic leukaemia (ALL). Several epidemiological studies have explored Greaves’ hypothesis by examining the link between various immune-stimulating exposures and the incidence of childhood cancers, particularly leukaemia. Factors such as vaginal birth,^31^ prolonged breastfeeding,^32^ early day-care attendance or exposure to infections^33 34^ and frequent vaccinations in childhood^35^ are considered potentially protective through immunological modulation. Moreover, maternal vaccinations during pregnancy may exert prenatal influences on the fetal immune system, potentially affecting the child’s cancer risk.^36 37^ In 2019, a population-based birth cohort study in Germany observed positive associations between several immunogenic exposures (eg, BCG vaccination, infections and immunoglobulin G titres).^38^ These findings suggest correlations between immunogenic exposures and unrelated antibody titres, which may underlie the nonspecific effects of vaccination on childhood morbidity and mortality and thus may play a role in modifying childhood cancer risk.

Despite substantial research, the link between vaccination and childhood cancer risk remains inconclusive, due to inconsistent results and often lacking adequate statistical power. Our previous meta-analysis found an inverse relationship between vaccination and leukaemia incidence and mortality.^39^ Specifically, BCG vaccination was associated with lower leukaemia-related mortality, after *Haemophilus influenzae* type b vaccination with decreased ALL incidence, and three or more vaccinations of any type with a lower risk of leukaemia and ALL. Our meta-analysis, which informed the development of the birth cohort described here, also highlighted key methodological gaps in previous studies, particularly inadequate exposure assessment and limited control for confounders such as infections. Therefore, the *German Million Children Cohort* should investigate in this first project the role of *Pre*natal and *Ch*ildhood *I*mmunizations and the Risk of Childhood *C*ancer (*PRECHIC*), by leveraging claims data from the *Ge*rman *P*h*a*rmacoepidemiological *R*esearch *D*atabase (GePaRD).

## Cohort description

### Data source and study population

*The German Million Children Cohort* is a historical birth cohort based on the health claims database *GePaRD* covering data from persons who have been insured with one of the four participating statutory health insurance providers since 2004 or later (~25 million persons). At present, the last available data year is 2022. In addition to demographic data, this database contains information on drug dispensations as well as outpatient (ie, from general practitioners and specialists) and inpatient services and diagnoses. Per data year, there is information on ~20% of the general population and all geographical regions of Germany are represented.^40 41^ Information on vaccination is obtained based on available codes of the German Uniform Assessment Standard (EBM). Diagnoses are coded according to the German modification of the International Classification of Diseases, 10th revision (ICD-10-GM).

The birth cohort includes all newborns in *GePaRD* born from 2004 to 2018. Further inclusion criteria were consistent information on sex and residence in Germany at birth. Due to data privacy, only the year of birth is available. Therefore, the start of the first insurance period was used as the cohort entry.

The cohort exit was defined as the earliest occurrence of any of the following events: cancer diagnosis, death, 31 December of the year the child turned 18, end of the current maximum observation period (31 December 2022) or termination of continuous insurance coverage.

### Outcome assessment: childhood cancer

The primary health outcome of the *PRECHIC* project in the *German Million Children Cohort* is childhood cancer. Cases were defined as individuals with at least one hospital discharge diagnosis within the ICD-10-GM category ‘C00-C97 — Malignant neoplasms’. Benign cancer diagnoses were not considered. We restricted the birth cohort to individuals born until 2018, ensuring that children born in 2018 may also have a follow-up period of up to 4 years (until the end of 2022).

The date of the first childhood cancer diagnosis was defined as either the date of hospital admission or the first outpatient cancer diagnosis, whichever occurred first.

### Exposure assessment: Vaccinations

Using claims data from *GePaRD*, individual vaccination histories for children since birth were assessed. The basic immunisation schedule that is recommended to be administered during the first 2 years of life by the *Ständige Impfkommission* (*STIKO*, responsible for vaccination guidelines in Germany) includes vaccinations against tetanus, diphtheria, pertussis, *H. influenzae* type b, poliomyelitis, hepatitis B, pneumococci, rotavirus, meningococci, measles, mumps, rubella and varicella.

Vaccination exposure variables include vaccination status (whether a child has ever been vaccinated or remained unvaccinated) and the completeness of vaccination coverage according to age-specific recommendations for both individual vaccines and overall immunisation schedule. Given that only a proxy for the date of birth was available and to account for potential vaccination delays due to factors such as illnesses (eg, frequent colds during winter months), a 6-month tolerance period was added to the *STIKO*-recommended time window for each vaccine. Vaccination was considered if they were within the recommended age window (plus the 6-month tolerance) and at least 14 days after any prior dose of the same vaccine. We also assessed whether there were vaccinations not included in the recommended basic vaccination scheme and the total number of administered shots for each vaccine.

For a subsample of the cohort in which data on the mother’s vaccination exposure during pregnancy were also available by linking the mother’s and the newborn’s data,^42^ we also ascertained vaccinations during pregnancy.

### Assessment of co-variables

To consider potential confounders regarding vaccinations and their impact on childhood cancer, we assessed dispensed medications (antibiotics) and we derived medical conditions like infections, syndromes predisposing to cancer (trisomy 21, ataxia-telangiectasia, Fanconi anaemia, Noonan syndrome, Beckwith-Wiedemann syndrome, Nijmegen breakage syndrome, Werner syndrome, Bloom syndrome, tuberous sclerosis) and immunological conditions (juvenile arthritis, lupus erythematosus, dermato-/polymyositis, systemic sclerosis, purpura anaphylactoides, Kawasaki disease, Takayasu syndrome, Panarteritis nodosa, Wegener’s granulomatosis, panarteritis, microscopic polyangiitis, Behçet’s disease, non-neuropathic hereditary familial amyloidosis, Crohn’s disease, ulcerative colitis). We used a previously developed algorithm that classifies the socioeconomic status (SES) based on information on the educational level. Children are assigned to the same SES as their parents.^43^ We classified the SES variable into two categories: (a) at least a secondary degree, indicating a higher SES and (b) no secondary degree, no degree or no information on the degree, indicating a lower SES.

### Utilisation of the birth cohort

The first research project in the German Million Children Cohort is to determine the association between prenatal and childhood immunisation and the risk of childhood cancer. The cohort analysis to answer this research question will be designed based on the principle of target trial emulation and will be described in detail in a separate publication. The target trial analyses will furthermore include sensitivity analyses to evaluate the robustness of key exposure definitions and assumptions. As a pre-work (see the Results section), we assessed the prevalence of childhood vaccination in the cohort, including both the recommended basic immunisations by the age of 2 years as well as optional vaccinations, and characterised incident cancer cases regarding age, type of cancer and geographical distribution. In the long term, this birth cohort may become a valuable resource for studying further research on childhood cancer, including survival and other outcomes.

### Patient and public involvement

Patients and the public were not involved in this study, as it used anonymised claims data.

## Findings to date

### Selection of the study population

**Figure 1:**
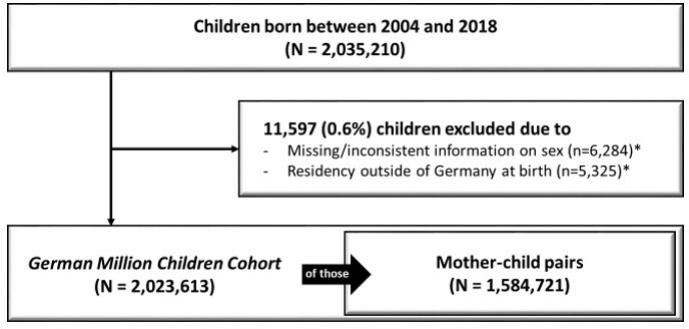
Flow diagram for building the *German Million Children Cohort* based on all children born between 2004 and 2018 in claims data of the *German Pharmacoepidemiological Research Database*. *12 children were assigned to both exclusion categories.

As shown in figure 1 2 035 210 children born from 2004 to 2018 were identified in *GePaRD*. A total of 6284 children were excluded due to missing or inconsistent information on sex, and 5325 due to residency outside Germany at birth. The final *German Million Children Cohort* included 2 023 613 children. Linkage to the mother’s data was possible for 1 584 721 (78.3%) of children.

**Figure 2:**
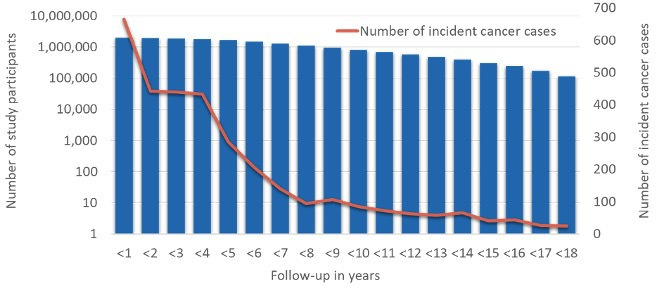
Distribution of the length of follow-up of children included in the *German Million Children Cohort* (born between 2004 and 2018, blue bars) and number of incident cancer cases (defined via International Classification of Diseases, 10th revision codes) by age (red line).

Overall, the *German Million Children Cohort* covers 17 752 995 person-years. Of the 2 023 613 children included in the cohort, 114 029 (5.6%) had a follow-up over the maximum follow-up period of 18 years (figure 2). The mean follow-up was 8.3 years. During follow-up, 3410 children were diagnosed with cancer (table 1).

**Table 1:** Sociodemographic factors of the children included in the *German Million Children Cohort*

Information	All children (n=2 023 613)		Children with maternal data (n=1 584 721)	
	Cancer cases (n=3410)	Children without cancer (n=2 020 203)	Cancer cases (n=2650)	Children without cancer (n=1 582 071)
Sex				
Male	1864 (54.7%)	1 036 213 (51.3%)	1429 (53.9%)	811 510 (51.3%)
Female	1546 (45.3%)	983 990 (48.7%)	1221 (46.1%)	770 561 (48.7%)
Year of birth				
2004	266 (7.8%)	108 247 (5.4%)	174 (6.6%)	71 607 (4.5%)
2005	257 (7.5%)	103 686 (5.1%)	196 (7.4%)	82 512 (5.2%)
2006	223 (6.5%)	103 610 (5.1%)	169 (6.4%)	80 884 (5.1%)
2007	222 (6.5%)	105 312 (5.2%)	179 (6.8%)	84 965 (5.4%)
2008	247 (7.2%)	118 110 (5.8%)	193 (7.3%)	85 702 (5.4%)
2009	250 (7.3%)	119 810 (5.9%)	203 (7.7%)	93 652 (5.9%)
2010	199 (5.8%)	119 990 (5.9%)	161 (6.1%)	96 216 (6.1%)
2011	259 (7.6%)	129 154 (6.4%)	195 (7.4%)	94 823 (6.0%)
2012	235 (6.9%)	136 196 (6.7%)	183 (6.9%)	106 532 (6.7%)
2013	215 (6.3%)	142 988 (7.1%)	178 (6.7%)	116 261 (7.3%)
2014	216 (6.3%)	150 328 (7.4%)	175 (6.6%)	121 022 (7.6%)
2015	220 (6.5%)	161 236 (8.0%)	175 (6.6%)	129 326 (8.2%)
2016	205 (6.0%)	171 653 (8.5%)	164 (6.2%)	137 222 (8.7%)
2017	216 (6.3%)	173 792 (8.6%)	175 (6.6%)	140 115 (8.9%)
2018	180 (5.3%)	176 091 (8.7%)	130 (4.9%)	141 232 (8.9%)
Length of follow-up (in years)				
Mean (SD)	4.33 (4.05)	8.30 (4.79)	4.26 (3.95)	8.21 (4.69)
Education of parent^43^				
High (at least secondary degree)	2665 (78.1%)	1 496 659 (74.1%)	2076 (78.3%)	1 176 816 (74.4%)
Low (no secondary degree, no degree or no information)	745 (21.9%)	523 544 (25.9%)	574 (21.7%)	405 255 (25.6%)
German index of socioeconomic deprivation (district of residence)^46^				
Low deprivation	924 (27.1%)	531 212 (26.3%)	699 (26.4%)	405 604 (25.6%)
Medium deprivation	1839 (53.9%)	1 146 416 (56.7%)	1436 (54.2%)	900 644 (56.9%)
High deprivation	638 (18.7%)	338 013 (16.7%)	510 (19.2%)	272 345 (17.2%)
Unknown	9 (0.3%)	4562 (0.2%)	5 (0.2%)	3478 (0.2%)

**Figure 3:**
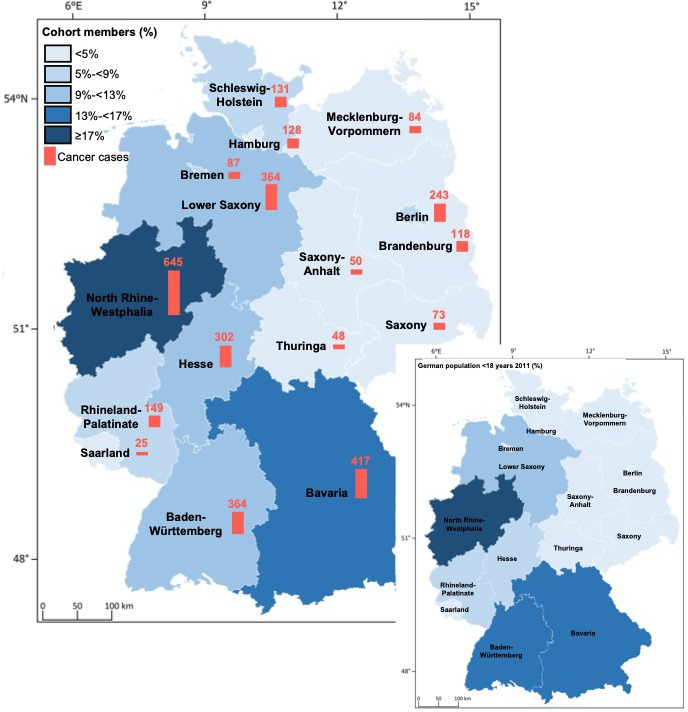
Distribution of children included in the *German Million Children Cohort* by residence at birth and cancer status across German states (2004–2018).

Figure 3 shows the distribution of included children in the *German Million Children Cohort* and of incident cancer cases across Germany’s federal states, with the highest number of cohort members coming from North Rhine-Westphalia, Bavaria, Baden-Württemberg and Lower Saxony.

**Figure 4:**
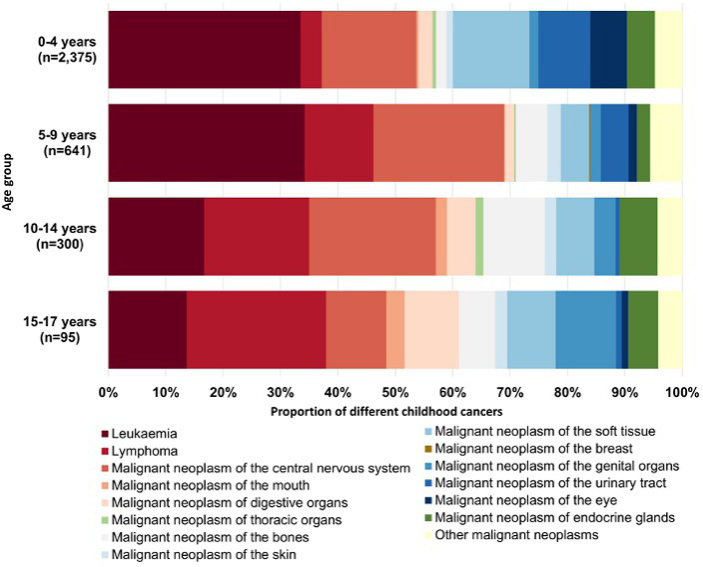
Distribution of childhood cancer cases by age group and cancer sites in the *German Million Children Cohort*, with comparison to the national population under 18 years (2011 census).

Leukaemia was the most common type of cancer, accounting for 31.6% of cases, with 77.1% of these being ALL. CNS tumours were the second most common type with a share of 18.0% (table 2, figure 4). Overall, 20.6% of all children in the cohort were hospitalised at least once during follow-up due to any infection (table 3). Additional sociodemographic and health-related details about the included children are provided in tables1  3.

**Table 2:** Childhood cancer sites in the *German Million Children Cohort*

Information	All cancer cases (n=3410)	Cancer cases with maternal data (n=2650)
*Cancer site (ICD-10, main discharge diagnosis*)		
Leukaemia	1077 (31.6%)	843 (31.8%)
ALL	830 (77.1%)	645 (76.5%)
B-cell ALL	13 (1.2%)	11 (1.3%)
AML	100 (9.3%)	84 (10.0%)
CML	4 (0.4%)	3 (0.4%)
Other leukaemia	130 (12.1%)	100 (11.9%)
Lymphoma	243 (7.1%)	187 (7.1%)
Non-Hodgkin’s lymphoma	162 (66.7%)	128 (68.4%)
Hodgkin’s lymphoma	81 (33.3%)	59 (31.6%)
Central nervous system	614 (18.0%)	467 (17.6%)
Mouth	17 (0.5%)	13 (0.5%)
Gastrointestinal organs	93 (2.7%)	72 (2.7%)
Thorax organs	20 (0.6%)	17 (0.6%)
Bones	117 (3.4%)	96 (3.6%)
Skin	50 (1.5%)	42 (1.6%)
Soft tissue	376 (11.0%)	292 (11.0%)
Breast	1 (0.0%)	1 (0.0%)
Genital organs	70 (2.1%)	52 (2.0%)
Urinary tract	249 (7.3%)	187 (7.1%)
Eyes	162 (4.8%)	122 (4.6%)
Endocrine glands	155 (4.5%)	124 (4.7%)
Others	167 (4.9%)	136 (5.1%)

ALL, acute lymphoblastic leukaemia; AML, acute myeloid leukaemia; CML, chronic myeloid leukaemia; ICD-10, International Classification of Diseases, 10th revision.

**Table 3:** Description of health-associated variables of included children in the *German Million Children Cohort*

Information	All children (n=2 023 613)		Children with maternal data (n=1 584 721)	
	Cancer cases (n=3410)	Children without cancer (n=2 020 203)	Cancer cases (n=2650)	Children without cancer (n=1 582 071)
*Any codes for vaccination-preventable infections (coded in the inpatient or outpatient setting*)				
No	3092 (90.7%)	1 831 720 (90.7%)	2414 (91.1%)	1 440 022 (91.0%)
Yes	318 (9.3%)	188 483 (9.3%)	236 (8.9%)	142 049 (9.0%)
Chickenpox	162 (50.9%)	117 351 (62.3%)	113 (47.9%)	86 134 (60.6%)
Diphtheria	1 (0.3%)	626 (0.3%)	1 (0.4%)	494 (0.3%)
*Haemophilus influenzae* type B infection	17 (5.3%)	4869 (2.6%)	14 (5.9%)	3857 (2.7%)
Hepatitis B	1 (0.3%)	709 (0.4%)	1 (0.4%)	535 (0.4%)
Measles	0 (0.0%)	1312 (0.7%)	0 (0.0%)	960 (0.7%)
Meningitis	3 (0.9%)	2308 (1.2%)	2 (0.8%)	1838 (1.3%)
Mumps	2 (0.6%)	991 (0.5%)	2 (0.8%)	758 (0.5%)
Pertussis	21 (6.6%)	12 412 (6.6%)	16 (6.8%)	8949 (6.3%)
Poliomyelitis	0 (0.0%)	102 (0.1%)	0 (0.0%)	73 (0.1%)
Pneumonia	5 (1.6%)	1449 (0.8%)	3 (1.3%)	1171 (0.8%)
Rota virus	120 (37.7%)	54 477 (28.9%)	98 (41.5%)	43 182 (30.4%)
Rubella	7 (2.2%)	2491 (1.3%)	3 (1.3%)	1890 (1.3%)
Tetanus	0 (0.0%)	167 (0.1%)	0 (0.0%)	116 (0.1%)
*Ever hospitalised for infections (main discharge diagnosis*)				
No	2704 (79.3%)	1 603 204 (79.4%)	2080 (78.5%)	1 249 944 (78.9%)
Yes	706 (20.7%)	416 999 (20.6%)	570 (21.5%)	334 127 (21.1%)
*Number of infection-related hospitalisations per year of follow-up*				
Mean (SD)	0.15 (0.98)	0.05 (1.14)	0.16 (1.04)	0.05 (1.20)
*Ever prescription of antibiotics*				
No	1274 (37.4%)	478 076 (23.7%)	983 (31.7%)	365 353 (23.1%)
Yes	2136 (62.6%)	1 542 127 (76.3%)	1667 (62.9%)	1 216 718 (76.9%)

### Vaccination status

**Figure 5:**
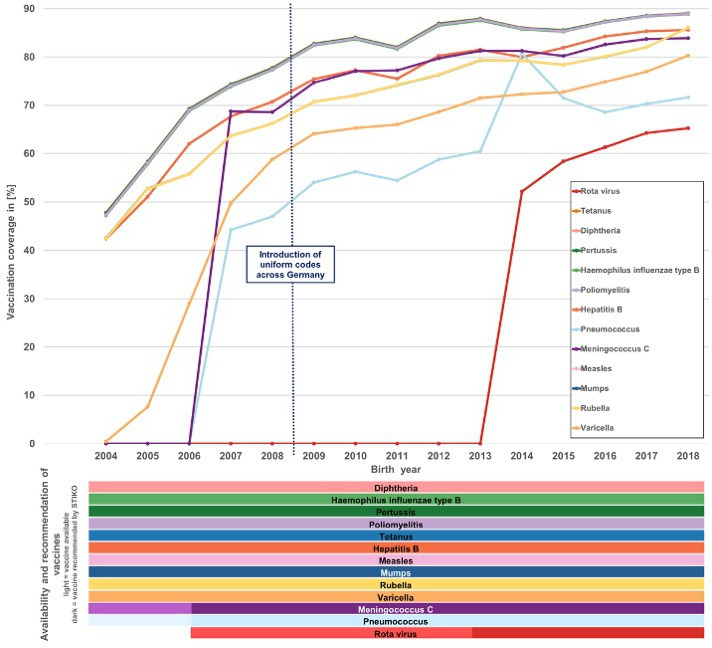
Proportion of completely vaccinated (according to recommendations of the *Ständige Impfkommission (STIKO*)) children by vaccine at age 30 months by birth year in the *German Million Children Cohort*.

The proportion of completely vaccinated children according to *STIKO* recommendations in Germany is shown in figure 5. The figure depicts the availability and recommendation periods for each vaccine. Notably, after the introduction of standardised national vaccination codes across Germany in 2008, a consistently high vaccination coverage of about 80% was observed (figure 5). The apparently lower vaccination coverage in previous years can best be explained by data gaps. The highest vaccination coverage was seen for vaccines against tetanus, diphtheria, *H. influenzae* type b, poliomyelitis and pertussis, reaching up to 89.0% in children born in 2018 (figure 5). The distribution of vaccination coverage across vaccines remained roughly similar since 2008 (figure 5).

More detailed information regarding the proportion of unvaccinated and incompletely vaccinated children, stratified by vaccine, birth cohort, cancer status and German federal states, is presented in online supplemental figures 1–6 and online supplemental tables 1–3). These figures provide a comprehensive overview, highlighting trends and variations across different birth years and vaccines.

Overall, a continuous increase in the proportion of completely and incompletely vaccinated children can be observed across the birth cohorts from 2004 to 2018. However, the introduction of new vaccines temporarily reduced the share of completely vaccinated children. For instance, following the 2004 varicella vaccination recommendation, only 0.5% of children born that year were completely vaccinated against varicella (online supplemental figure 1), resulting in only 0.2% being completely vaccinated overall (online supplemental figure 4). Coverage improved steadily in subsequent years.

However, it should be noted that vaccination estimates for the earliest birth cohorts may be affected by incomplete coding in the initial years of data availability, which could lead to an underestimation of true vaccination coverage during this period. A similar decline occurred after the 2013 introduction of rotavirus vaccination, with full vaccination rates dropping from 50.5% to 40.8%, then gradually recovering to 48.9% by 2018. Despite these fluctuations, the combined rate of full and partial vaccination remained stable at around 94% (online supplemental figure 4). Possible explanations for the initially low vaccination coverage following the introduction of new vaccines could include a lack of awareness among parents or proactive recommendations by physicians, parental concerns about potential side effects and a low perceived severity of the disease based on prior personal or community experiences. Contrary to that, we see that the change in the recommendation for pneumococcus vaccination from four to three shots in 2015 resulted in a sudden increase in the proportion of completely vaccinated children at age 30 months in the 2014 birth cohort (online supplemental figure 3).

Vaccination coverage varied between German federal states (online supplemental figure 5 and 6). The highest proportions of completely vaccinated children were observed in Mecklenburg-Vorpommern (49.5%), Saxony-Anhalt (47.5%) and Brandenburg (46.1%), the lowest proportions of completely vaccinated children were recorded in Bavaria (32.1%), Hamburg (31.9%) and Saxony (13.8%, (online supplemental figure 5). The highest proportions of unvaccinated children were found in Baden-Württemberg (17.8%), Berlin (17.0%) and Hamburg (16.3%), the lowest proportions of unvaccinated children were observed in Bremen (4.4%), Saxony-Anhalt (4.3%) and Mecklenburg-Vorpommern (3.9%, online supplemental figure 6). These figures summarise the full observation period and therefore include early years in which vaccination coding may have been incomplete, which could contribute to an underestimation of coverage, particularly for the earliest cohorts.

Among the 1 584 721 children in whom information from pregnancy was available through linkage to mother’s data, 7.7% were born to mothers who received at least one vaccination during pregnancy (table 4).

**Table 4:** Prenatal immunisation of children with available maternal data in the *German Million Children Cohort*

Information	Children with maternal data (n=1 584 721)	
	Cancer cases (n=2650)	Children without cancer (n=1 582 071)
*Ever vaccinated during pregnancy*	2414 (91.1%)	1 440 022 (91.0%)
No	2468 (93.1%)	1 460 628 (92.3%)
Yes	182 (6.9%)	121 443 (7.7%)
First trimester	47 (25.8%)	33 179 (27.3%)
Second trimester	96 (52.7%)	58 559 (48.2%)
Third trimester	45 (24.7%)	33 488 (27.6%)
*Vaccination category (based on STIKO recommendations*)		
Recommended vaccines	167 (91.8%)	110 007 (90.6%)
Influenza	154 (84.6%)	100 516 (82.8%)
Pertussis	16 (8.8%)	13 146 (10.8%)
Acceptable vaccines	28 (15.4%)	20 696 (17.0%)
Diphtheria	22 (12.1%)	17 923 (14.8%)
Tetanus	25 (13.7%)	19 046 (15.7%)
Poliomyelitis	12 (6.6%)	8744 (7.2%)
Hepatitis A	2 (1.1%)	902 (0.7%)
Hepatitis B	1 (0.5%)	1266 (1.0%)
Contraindicated vaccines	1 (0.5%)	1729 (1.4%)
Measles	1 (0.5%)	1393 (1.1%)
Mumps	1 (0.5%)	1363 (1.1%)
Rubella	1 (0.5%)	1565 (1.3%)
Varicella	0 (0.0%)	221 (0.2%)

STIKO, Ständige Impfkommission.

## Strengths and limitations

To our knowledge, this is the first historical birth cohort study in Germany leveraging claims data to investigate the relationship between immunisations and the risk of childhood cancers. The large size of the *German Million Children Cohort* will also allow us to assess rare childhood cancer sites and to stratify by vaccination type.

Using routinely collected claims data for vaccinations avoids recall or non-responder bias, while comprehensive information on comorbidities and medications allows adjustment for important confounders.

The long follow-up ensures the detection of childhood cancers occurring later in childhood or adolescence. Furthermore, linking about 78% of children with the mother’s data during pregnancy may also allow assessment of prenatal factors, such as maternal vaccinations and their influence on childhood cancer risk.

Nevertheless, we acknowledge certain limitations in our cohort. The assessment of vaccinations lacks completeness before 2008, prior to the nationwide implementation of standardised EBM billing codes. This potential misclassification affecting children born before 2008 is expected to be non-differential and will be considered in the interpretation of the study’s findings. For cancer outcomes, only inpatient diagnoses were included, as in the German healthcare system, childhood cancer cases are routinely handled in hospital settings. Although these codes ensure high validity, ICD-10 codes have limited information on histology or molecular cancer characteristics. Additionally, claims data provide limited information on lifestyle and environmental exposures. Private health insurance is not covered in the database and the population insured by the AOK, which tends to have a lower SES, is under-represented in *GePaRD*. Since SES of the cohort members is defined based on the highest parental educational attainment, we compared the cohort distribution to national statistics. In Germany, 79% of adults aged 35–44 years, an age group representative of potential parents of cohort participants, hold a secondary school qualification and would therefore be classified in the higher SES category.^44^ In our cohort, 78% of participants are classified in this category, indicating close alignment with the national distribution. While this is not directly relevant regarding internal validity of assessing the impact of vaccinations on childhood cancer in the *PRECHIC* project, it should be considered in the interpretation of descriptive results such as vaccination uptake or cancer incidences (table 1).

## Future perspectives

The *German Million Children Cohort* represents a significant resource for advancing our understanding of childhood cancer. Large-scale cohort infrastructures are increasingly recognised as essential for addressing complex aetiologic questions in paediatric populations.^45^ The comprehensive data available in our cohort enables future exploration of additional factors influencing cancer and other diseases in childhood, and the extension of the observation period will allow us to extend the study’s scope further.

## Supplementary material

10.1136/bmjopen-2025-113411 online supplemental file 1

10.1136/bmjopen-2025-113411 online supplemental file 2

## Data Availability

No data are available.
