# Supplementary material for: German Million Children Cohort: a historical birth cohort based on claims data to investigate the impact of immunisation and other early life factors on the risk of cancer and other diseases in childhood – cohort profile
Source: BMJ Open. 2026 Mar 26;16(3):e113411. doi: 10.1136/bmjopen-2025-113411 (PMC13034273; doi:10.1136/bmjopen-2025-113411)
Supplement: online supplemental file 1 [file bmjopen-16-3-s001.docx]

**Supplementary Material**

# Cohort profile: The *German Million Children Cohort* –

# A historical birth cohort based on claims data

Lara Kim Brackmann^1^, Loviisa Mulanje^1,2^, Bianca Kollhorst^3^, Sophie Langbein^3^, Ulrike Haug^2,4^, Wolfgang Ahrens^1^, Rajini Nagrani^1^, Manuela Marron^1^

^1^ Epidemiological Methods and Etiological Research, Leibniz Institute for Prevention Research and Epidemiology – BIPS, Bremen, Germany

^2^ Faculty of Human and Health Sciences, University of Bremen, Bremen, Germany

^3^ Biometry and Data Management, Leibniz Institute for Prevention Research and Epidemiology – BIPS, Bremen, Germany

^4^ Clinical Epidemiology, Leibniz Institute for Prevention Research and Epidemiology – BIPS, Bremen, Germany

**
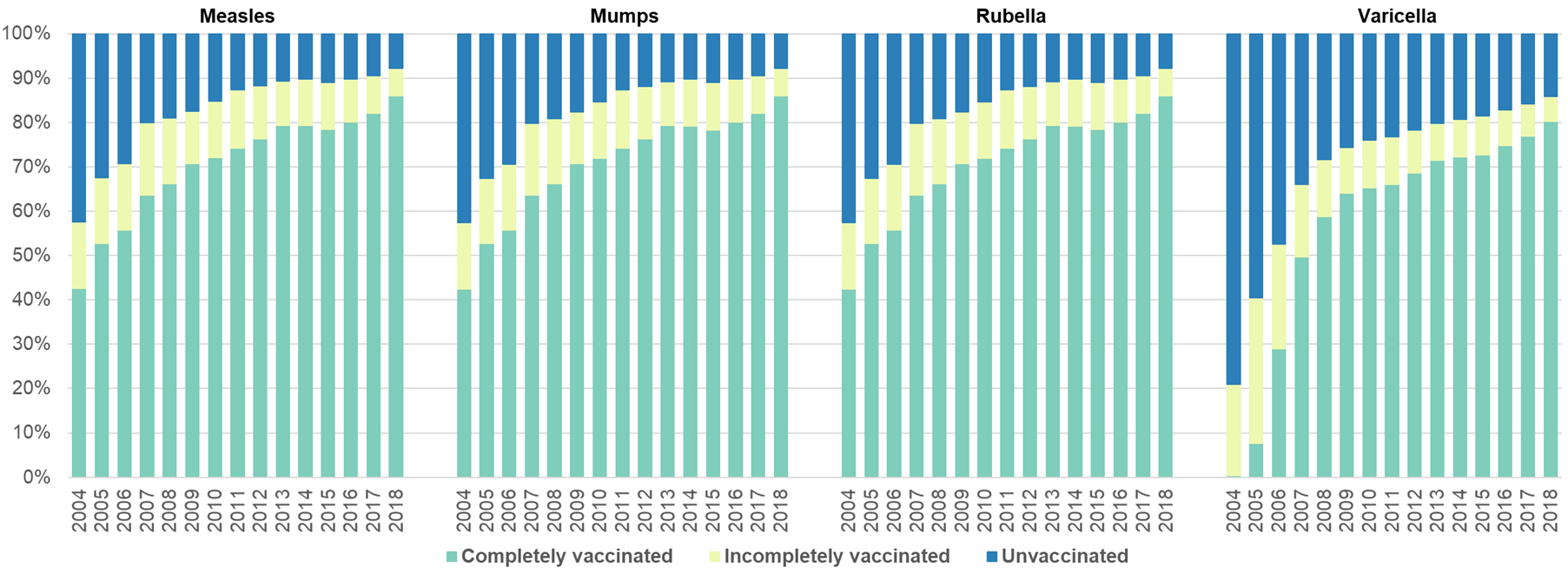
**

**Supplementary Figure 1:** Vaccination against measles, mumps, rubella, and varicella (MMRV) vaccination - Proportion of completely, incompletely and unvaccinated children (according to recommendations of the *STIKO* (*Ständige Impfkomission*)) at age 30 months by birth year in the *German Million Children Cohort*. Abbreviations: Measles, mumps, rubella, and varicella, MMRV; *Ständige Impfkomission, STIKO*.


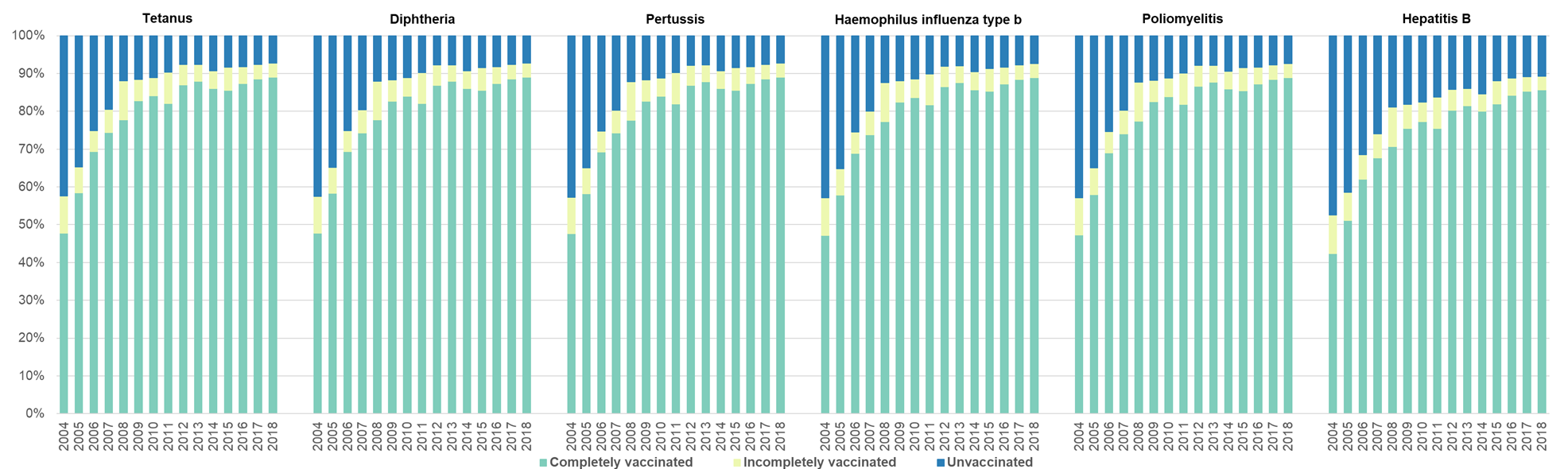


**Supplementary Figure 2:** Sixfold vaccination - Proportion of completely, incompletely and unvaccinated children (according to recommendations of the *STIKO* (*Ständige Impfkomission*)) at age 30 months by birth year in the *German Million Children Cohort* (n=2,022,122. Abbreviation: *Ständige Impfkomission, STIKO*.


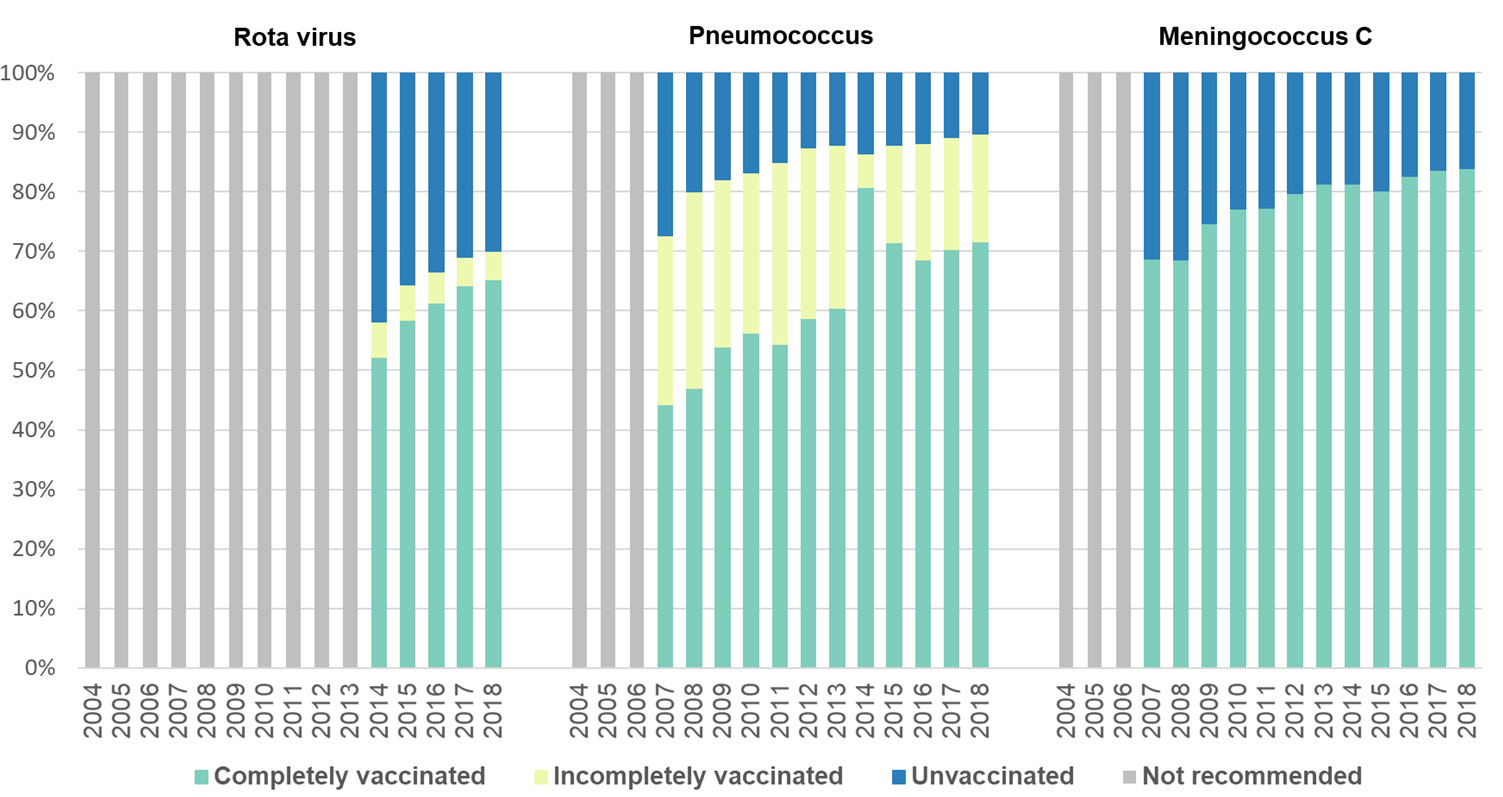


**Supplementary Figure 3:** Other recommended vaccinations - Proportion of completely, incompletely and unvaccinated children (according to recommendations of the *STIKO* (*Ständige Impfkomission*)) at age 30 months by birth year in the *German Million Children Cohort*. Abbreviation: *Ständige Impfkomission, STIKO*.


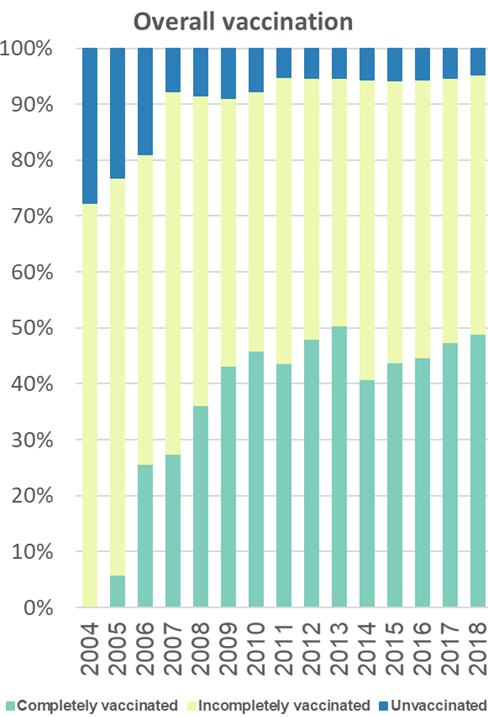


**Supplementary Figure 4:** Proportion of completely, incompletely and unvaccinated children according to all recommended vaccinations of the *STIKO* (*Ständige Impfkomission*)) at age 30 months by birth year in the *German Million Children Cohort*. Abbreviation*: Ständige Impfkomission, STIKO*.

**
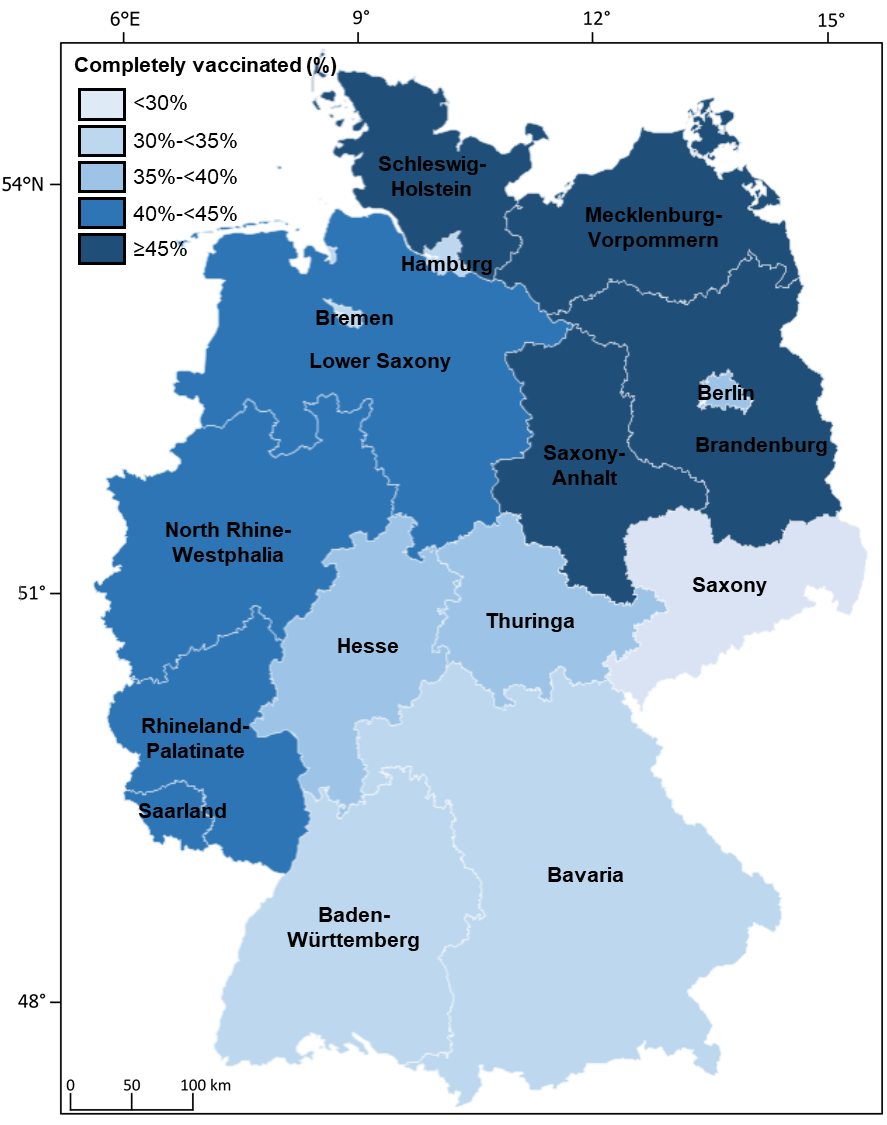
**

**Supplementary Figure 5:** Proportion of completely vaccinated children according to all recommended vaccinations of the *STIKO* (*Ständige Impfkomission*)) at age 30 months in the *German Million Children Cohort* by German federal state. Abbreviation: *Ständige Impfkomission, STIKO*.


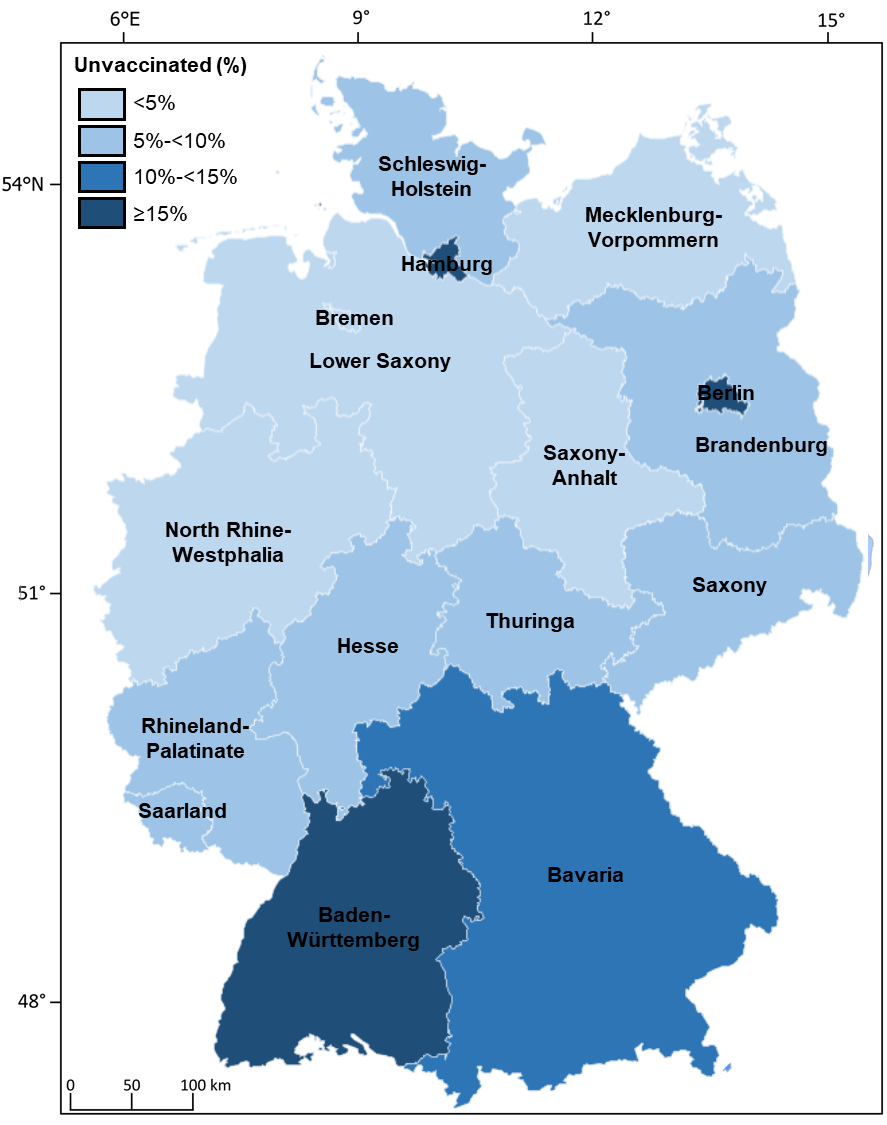


**Supplementary Figure 6:** Proportion of unvaccinated children according to all recommended vaccinations of the *STIKO* (*Ständige Impfkomission*)) at age 30 months in the *German Million Children Cohort* by German federal state. Abbreviation: *Ständige Impfkomission, STIKO*.
