# Supplementary material for: German Million Children Cohort: a historical birth cohort based on claims data to investigate the impact of immunisation and other early life factors on the risk of cancer and other diseases in childhood – cohort profile
Source: BMJ Open. 2026 Mar 26;16(3):e113411. doi: 10.1136/bmjopen-2025-113411 (PMC13034273; doi:10.1136/bmjopen-2025-113411)
Supplement: Supplementary file 3 [file bmjopen-2025-113411.reviewer_comments.pdf]

PEER REVIEW HISTORY

BMJ Open publishes all reviews undertaken for accepted manuscripts. Reviewers are asked to complete a checklist review form and are provided with free text boxes to elaborate on their assessment. These free text comments are reproduced below.

ARTICLE DETAILS

Title (Provisional)

Cohort profile: The German Million Children Cohort -A historical birth cohort based on claims data to investigate the impact of immunisation and other early life factors on the risk of cancer and other diseases in childhood

Authors

Brackmann, Lara Kim; Mulanje, Loviisa; Langbein, Sophie; Kollhorst, Bianca; Haug, Ulrike; Ahrens, Wolfgang; Nagrani, Rajini; Marron, Manuela

---

VERSION 1 - REVIEW

**Reviewer**	**1**
**Name**	**Uwishema, Olivier**
**Affiliation**	**Oli Health Magazine Organization Kigali**
**Date**	**20-Jan-2026**
**COI**	**None**

Comments to the Authors

Dear Authors,

Thank you so much for the opportunity to review the manuscript ‘’ bmjopen-2025-113411 ‘’ titled ‘’ Cohort profile: The German Million Children Cohort -A historical birth cohort based on claims data to investigate the impact of immunization and other early life factors on the risk of cancer and other diseases in childhood ‘’

Recommendation: Major revision

Major comments (need to be carefully addressed)

1. **Target trial emulation needs substantially more specification**
   You state the PRECHIC analysis “will be designed based on the principle of target trial emulation,” but the manuscript does not yet provide the core components readers need to judge causal validity (e.g., explicit eligibility criteria, “time zero,” treatment

strategies, assignment procedures, follow-up definition, causal contrast/estimand, handling of censoring, and planned statistical approach).

Suggestion: Add a concise "Target trial protocol" table aligned to Hernán & Robins style (eligibility, strategies, assignment, follow-up, outcome, estimand, analysis plan) and explicitly address common biases in vaccine studies (immortal time, time-varying exposure, confounding by healthcare utilization and infection history).

2. **Exposure definition: clarify operational rules and implications of limited birth-date granularity**
   You appropriately note that only year of birth is available and therefore use insurance start as cohort entry.

   You then apply a **six-month tolerance** around STIKO windows to define "on-time/completeness."

   Suggestions:

   - Explain how the "proxy date of birth" is constructed in practice and how misclassification is expected to vary by calendar year and by vaccine schedule timing.
   - Provide sensitivity analyses you plan to run (e.g., alternative tolerance windows; restricting to birth cohorts after standardized coding; analyses based on "ever vaccinated" vs "age-appropriate completeness").

3. **Outcome definition: potential under-ascertainment and limited clinical detail**
   Cancer cases are defined based on **hospital discharge diagnosis** in "C00–C97," with date as admission or first outpatient diagnosis, and you acknowledge limited characterization (ICD-10 codes without histology/molecular detail).

   Suggestions:

   - Clarify how outpatient cancer diagnoses are used if the primary case definition relies on hospital discharge diagnoses (this reads slightly inconsistent).
   - Add validation references or rationale for sensitivity/specificity of inpatient-only ascertainment in GePaRD (if available).
   - State whether benign/borderline CNS tumors are excluded and how relapse/second cancers are handled.

4. **Confounding control: strengthen measurement strategy and limitations**
   You mention assessing antibiotic dispensations and multiple medical conditions

(infections, cancer-predisposition syndromes, immunological conditions) and parental-education-based SES (collapsed to higher vs lower).

Suggestions:

- Explain timing rules (baseline vs time-updated covariates), especially for infections and antibiotics which may be mediators or confounders depending on the causal question.
- Consider adding proxies for healthcare-seeking behavior (well-child visits, preventive checkups, vaccination provider type) to reduce confounding by utilization.

5. **Generalizability and descriptive estimates: expand discussion**
   You note underrepresentation of **AOK (lower SES)** and absence of private insurance, and that pre-2008 vaccination coding is incomplete.

   Suggestions:

   - Present a clearer comparison of cohort demographics vs national statistics, especially SES and geography.
   - For descriptive vaccination coverage and incidence maps, emphasize that estimates for early years likely reflect coding artifacts (your Figure 5 discussion begins to do this).

6. **Collaboration/data access section: good start, but make it more actionable**
   The data sharing statement is clear that external access requires collaboration and on-premises BIPS work with a guest researcher agreement.

   Suggestions:

   - Add what types of collaborations are encouraged (methods development, disease-specific analyses, international comparators), a point of contact, and the typical steps/timelines for proposing a project.

7. **Incorporating the very latest published articles**

The below article is very relevant for citation in your paper:

(1) Uwishema, O., Frederiksen, K. S., Badri, R., Pradhan, A. U., Shariff, S., Adanur, I., Dost, B., Esene, I., & Rosseau, G. (2023). Epidemiology and etiology of brain cancer in Africa: A systematic review. *Brain and behavior*, *13*(9), e3112. https://doi.org/10.1002/brb3.3112

Relevance:

- Directly relevant to:

    - childhood and brain cancer epidemiology
    - etiological uncertainty
- Strengthens rationale for:
    - large cohort studies
    - population-based cancer surveillance

Where it should be cited: It can be cited in the **introduction to highlight the** gaps in understanding childhood brain cancer etiology

I can also be cited in the **discussion** to highlight the need for large-scale cohorts to address etiologic questions.

Minor comments (editorial/clarity)

- In the introduction, there is a duplication: "inconclusive, inconclusive" should be corrected.

- Clarify wording around "Vaccination were considered…" (grammar) and ensure consistent terminology (immunisation vs immunization).

- Figures are informative (flow diagram, follow-up distribution, Germany map, vaccine uptake trends). Consider adding figure captions that explicitly note key denominators and time windows (e.g., "at age 30 months") to improve standalone interpretability.

VERSION 1 - AUTHOR RESPONSE

Reviewer: 1

Dr. Olivier Uwishema, Oli Health Magazine Organization Kigali

Comments to the Author:

Dear Authors,

Thank you so much for the opportunity to review the manuscript '' bmjopen-2025-113411 '' titled '' Cohort profile: The German Million Children Cohort -A historical birth cohort based on claims data to investigate the impact of immunization and other early life factors on the risk of cancer and other diseases in childhood ''

Recommendation: Major revision

Major comments (need to be carefully addressed)

Dear Dr. Uwishema,

we sincerely thank you for your careful and thoughtful evaluation of our manuscript. We are impressed by how thoroughly you have engaged with the complexity of our data structure and by the many insightful and highly relevant points you have raised for considering our research question. We fully agree with your comments and greatly appreciate the constructive nature of your suggestions. In the following point-by-point response, we address each of your comments in detail.

Before doing so, we would like to note that we are currently preparing a separate manuscript describing the target trial mentioned in the here presented cohort profile. This forthcoming paper is intended to comprehensively address many of the methodological aspects and improvements you suggested. We believe that incorporating the full target trial framework into the present cohort profile would substantially increase its complexity and scope beyond what is appropriate for this manuscript. To maintain clarity and avoid redundancy, we therefore prefer to present the target trial methods in detail in the dedicated manuscript.

In this context, we would be very pleased to suggest you as a potential reviewer for the target trial manuscript, as your expertise and perspective would be highly valuable.

1. Target trial emulation needs substantially more specification

You state the PRECHIC analysis "will be designed based on the principle of target trial emulation," but the manuscript does not yet provide the core components readers need to judge causal validity (e.g., explicit eligibility criteria, "time zero," treatment strategies, assignment procedures, follow-up definition, causal contrast/estimand, handling of censoring, and planned statistical approach).

Suggestion: Add a concise "Target trial protocol" table aligned to Hernán & Robins style (eligibility, strategies, assignment, follow-up, outcome, estimand, analysis plan) and explicitly address common biases in vaccine studies (immortal time, time-varying exposure, confounding by healthcare utilization and infection history).

You raise an important point regarding the need for a clear specification of the target trial framework. The components you describe are the central focus of the above mentioned separate manuscript we are currently preparing, which will provide a comprehensive description of the target trial, including methodological details and bias considerations.

We believe, including this full framework in the present cohort profile would extend its scope beyond what is appropriate for this format and would lead to redundancy across publications. We have therefore now mentioned in the manuscript that the detailed target trial specification will be presented in the dedicated paper.

2. Exposure definition: clarify operational rules and implications of limited birth-date granularity

You appropriately note that only year of birth is available and therefore use insurance start as cohort entry.

You then apply a six-month tolerance around STIKO windows to define "on-time/completeness."

Suggestions:

- Explain how the "proxy date of birth" is constructed in practice and how misclassification is expected to vary by calendar year and by vaccine schedule timing.
- Provide sensitivity analyses you plan to run (e.g., alternative tolerance windows; restricting to birth cohorts after standardized coding; analyses based on "ever vaccinated" vs "age-appropriate completeness").

Regarding the proxy date of birth, we defined it as the start date of insurance within the recorded birth year, as described in the Methods section. In Germany, children are typically insured very shortly after birth, as several preventive examinations take place within the first weeks of life that are covered through statutory health insurance. We acknowledge that children born at the very end of a calendar year may only appear in the database at the beginning of the following year. To address this, we implemented a grace period in the subsequent year to capture these cases appropriately. The operational details of this procedure are described in the Methods section. Overall, we expect any remaining misclassification to be minimal with respect to vaccination status.

In addition, you raise a valuable point regarding the importance of sensitivity analyses around the tolerance window definition. Within the target trial framework, we plan to conduct several sensitivity analyses that extend beyond the timing of individual vaccinations. These will include alternative definitions of vaccination completeness, such as receipt of a full primary immunization series within the first two years of life, as well as analyses that account for the occurrence of infections.

Because these analyses are embedded in the target trial design, they will be described and justified in detail in the dedicated manuscript. We will clarify this in the cohort profile to make the planned analytical scope transparent.

3. Outcome definition: potential under-ascertainment and limited clinical detail

Cancer cases are defined based on hospital discharge diagnosis in "C00–C97," with date as admission or first outpatient diagnosis, and you acknowledge limited characterization (ICD-10 codes without histology/molecular detail).

Suggestions:

- Clarify how outpatient cancer diagnoses are used if the primary case definition relies on hospital discharge diagnoses (this reads slightly inconsistent).
- Add validation references or rationale for sensitivity/specificity of inpatient-only ascertainment in GePaRD (if available).
- State whether benign/borderline CNS tumors are excluded and how relapse/second cancers are handled.

You point out an important need for clarification regarding the cancer case definition. In the German healthcare system, children with cancer are routinely managed in hospital settings and therefore receive at least one coded inpatient diagnosis. We verified this assumption through manual review of several sampled cases. Outpatient diagnoses were incorporated only when they preceded the first hospital diagnosis, allowing us to assign an earlier and more accurate diagnosis date in those situations. We will revise the manuscript to clarify this workflow and avoid the impression of inconsistency. At present, there is no published validation study specifically quantifying the sensitivity or specificity of inpatient cancer ascertainment in GePaRD, but we described the rationale for our approach based on the structure of paediatric oncology care in Germany.

For the currently planned analyses, benign tumours were excluded. We now stated this explicitly in the manuscript. The cohort follow-up for cancer cases ends at the first diagnosis. Therefore, second cancers are not included in the present cohort. However, we have developed an algorithm to identify relapses and second primary tumours within the cohort for future research questions.

4. Confounding control: strengthen measurement strategy and limitations

You mention assessing antibiotic dispensations and multiple medical conditions (infections, cancer-predisposition syndromes, immunological conditions) and parental-education-based SES (collapsed to higher vs lower).

Suggestions:

- Explain timing rules (baseline vs time-updated covariates), especially for infections and antibiotics which may be mediators or confounders depending on the causal question.
- Consider adding proxies for healthcare-seeking behavior (well-child visits, preventive checkups, vaccination provider type) to reduce confounding by utilization.

You make an excellent point regarding the timing and definition of covariates. In our planned target trial, we have defined both baseline and time-updated covariates, and, as mentioned above, we will conduct a sensitivity analysis specifically for infections to assess their potential role in the pathway. Additionally, we have created a variable capturing healthcare contact, which includes attendance at recommended preventive checkups, to account for differences in healthcare utilization. These elements will be described in detail in the target trial manuscript.

5. Generalizability and descriptive estimates: expand discussion

You note underrepresentation of AOK (lower SES) and absence of private insurance, and that pre-2008 vaccination coding is incomplete.

Suggestions:

- Present a clearer comparison of cohort demographics vs national statistics, especially SES and geography.
- For descriptive vaccination coverage and incidence maps, emphasize that estimates for early years likely reflect coding artifacts (your Figure 5 discussion begins to do this).

You raise an important point about contextualizing the cohort with respect to national demographics and potential coding artifacts. To better illustrate representativeness, we have expanded Figure 3 to include the percentage distribution of individuals under 18 years by federal state in the general population, allowing a direct comparison with our cohort. We have also added a section comparing our socioeconomic status measure, based on the highest parental educational attainment, with German national educational statistics for adults aged 35–44 years, an age group representative for potential parents of cohort participants. This comparison indicates that the cohort reflects the national distribution in principal.

Regarding vaccination coverage and incidence patterns in earlier years, we have added clarifying statements in the corresponding text sections to emphasize that estimates from the early period are likely influenced by incomplete coding and should be interpreted with caution.

6. Collaboration/data access section: good start, but make it more actionable

The data sharing statement is clear that external access requires collaboration and on-premises BIPS work with a guest researcher agreement.

Suggestions:

- Add what types of collaborations are encouraged (methods development, disease-specific analyses, international comparators), a point of contact, and the typical steps/timelines for proposing a project.

Thank you for your comment. The regulations regarding data protection are very strict. Therefore, it is difficult to gain access to the data. In exceptional cases, third parties may receive permission to do so. However, as this involves contractual agreements, the administrative process involved is demanding and time intensive and therefore needs to be discussed on a case by case basis. Remote access is strictly prohibited.

7. Incorporating the very latest published articles

The below article is very relevant for citation in your paper:

- Uwishema, O., Frederiksen, K. S., Badri, R., Pradhan, A. U., Shariff, S., Adanur, I., Dost, B., Esene, I., & Rosseau, G. (2023). Epidemiology and etiology of brain cancer in Africa: A systematic review. Brain and behavior, 13(9), e3112. https://doi.org/10.1002/brb3.3112

Relevance:

- Directly relevant to:
  - childhood and brain cancer epidemiology
  - etiological uncertainty
- Strengthens rationale for:
  - large cohort studies
  - population-based cancer surveillance
- Where it should be cited: It can be cited in the introduction to highlight the gaps in understanding childhood brain cancer etiology
- I can also be cited in the discussion to highlight the need for large-scale cohorts to address etiologic questions.

Thank you for this helpful suggestion. We have cited your recent work in the Future Perspectives section to highlight the importance of large-scale cohort infrastructures for addressing complex etiologic questions in pediatric research. We believe this reference strengthens the contextual framing of the cohort's long-term scientific value.

Minor comments (editorial/clarity)

- In the introduction, there is a duplication: "inconclusive, inconclusive" should be corrected.

Thank you for mentioning this, we changed this.

- Clarify wording around "Vaccination were considered…" (grammar) and ensure consistent terminology (immunisation vs immunization).

Thank you, we corrected the wording and aligned the terminology.

- Figures are informative (flow diagram, follow-up distribution, Germany map, vaccine uptake trends). Consider adding figure captions that explicitly note key denominators and time windows (e.g., "at age 30 months") to improve standalone interpretability.

This is a good point; we added more informative descriptions to our Figures.

VERSION 2 - REVIEW

**Reviewer**	**1**
**Name**	**Uwishema, Olivier**

Affiliation **Oli Health Magazine Organization Kigali**

Date **09-Mar-2026**

COI

Many thanks to the authors, and congratulations! All comments and suggestions from my end have been meticulously addressed and incorporated into the current version of the paper. Each critique was carefully evaluated, and corresponding modifications were made to enhance the scholarly quality, scientific rigor, and overall presentation of the research. The manuscript now meets academic standards and publication criteria. After a thorough assessment, I confidently recommend that this revised version is fully prepared and suitable for publication in BMJ Open.
